# Physicochemical and Photocatalytic Properties under Visible Light of ZnO-Bentonite/Chitosan Hybrid-Biocompositefor Water Remediation

**DOI:** 10.3390/nano12010102

**Published:** 2021-12-29

**Authors:** Imane Aadnan, Omar Zegaoui, Abderrahim El Mragui, Joaquim Carlos Gomes Esteves da Silva

**Affiliations:** 1 Research Team “Materials and Applied Catalysis: MCA”, CBAE Laboratory, URL-CNRST N°13, Faculty of Sciences, Moulay Ismail University of Meknes, P.O. Box 11201 Zitoune, Meknès 50700, Morocco; i.aadnan@edu.umi.ac.ma (I.A.); a.elmragui@edu.umi.ac.ma (A.E.M.); 2 (UP)—Research Center in Chemistry, DGAOT, Faculty of Sciences, University of Porto, Rua do Campo Alegre, s/n, 4169-007 Porto, Portugal; jcsilva@fc.up.pt

**Keywords:** ZnO-bentonite/chitosan, hybrid-biocomposite, photocatalysis, methyl orange, visible light, mechanism

## Abstract

In this investigation, a hybrid-biocomposite “ZnO-Bentonite/Chitosan” was synthesized using inexpensive and environmentally friendly materials (Bentonitechitosan) and (ZnO). It was used as a photocatalyst for water remediation. The structural, optical, thermal, and morphological properties of the synthesized hybrid-biocomposite were investigated using XRD, FTIR spectroscopy, UV-vis diffuse reflectance spectroscopy, TGA, XPS, and SEM-EDS. The thermal measurements showed that the decomposition of CS was postponed progressively by adding PB and ZnO, and the thermal stability of the synthesized hybrid-biocomposite was improved. The characterization results highlighted strong interactions between the C–O, C=O, -NH_2_, and OH groups of chitosan and the alumina-silica sheets of bentonite on the one side, and between the functional groups of chitosan (-NH_2_, OH) and ZnO on the other side. The photocatalytic efficiency of the prepared hybrid-biocomposite was assessed in the presence of Methyl Orange (MO). The experiments carried out in the dark showed that the MO removal increased in the presence of Zn-PB/CS hybrid-biocomposite (86.1%) by comparison with PB (75.8%) and CS (65.4%) materials. The photocatalytic experiments carried out under visible light showed that the MO removal increased 268 times in the presence of Zn-PB/CS by comparison withZnO.The holes trapping experiments indicated that they are the main oxidative active species involved in the MO degradation under both UV-A and visible light irradiations.

## 1. Introduction

Water consumption is continuously increasing due to water demand in the agricultural and industrial sectors as well as in domestic utilities. It has been reported that about two million tons of wastewater coming from agriculture and industrial plants are daily discharged into the world’s natural water resources [1]. This inevitably leads to a drastic reduction of water reserves. Worse, the scarcity of water resources is accentuated by the increased desertification, particularly in Mediterranean countries. To meet the challenge of water supply, wastewater treatment plants should be installed, and the reutilization of treated wastewater should be considered as an additional water resourcein these countries’ waterpolicies. In this regard, several wastewater treatment processes are currently used such as coagulation/flocculation, ozonation, adsorption, and Advanced Oxidation processes (AOPs) [1,2,3,4]. The AOPs have proven their effectiveness as innovative wastewater treatment technologies. These processes are based on the insitu generation of highly reactive transitory species (i.e., HO^•^, O_2_^•−^, e^−^) for refractory organic compounds’ mineralization [4]. Among the AOPs, heterogeneous photocatalysis has demonstrated high efficiency in degrading a large number of ambiguous refractory organics into readily biodegradable compounds [1,5,6]. The heterogeneous photocatalyticmechanism relieson the absorption of photons with energy able to excite an electron in valence band (VB) to conduction band (CB), thus generating electron/hole pairs with reductive/oxidative capacity (Equation (1)). This step is followed by a migration of the charge carriers to the surface of the semiconductor particles in order to initiate redox reactions (Equation (2)). The holes can react with adsorbed water to form hydroxyl radicals (HO^•^) (Equations (3) and (4)) while the electrons react with the adsorbed oxygen to give superoxide radicals O_2_^•−^ (Equation (5)). Among the various used photocatalysts [7,8,9], ZnO pure or mixed with other materials has attracted special attention due to its good physicochemical, photocatalytic, antibacterial, and antifungal properties, its non-toxicity, its facile and low-cost production, and so forth [5,7,10,11,12,13,14]. However, like various metal oxide semiconductors, the photocatalytic effectiveness of ZnO is limited to the UV region (Band gap energy of ZnO= 3.30–3.32 eV [9]). Furthermore, potential recycling of pure ZnO nanoparticles is very difficult and a significant amount of material is lost during the recovery process. In order to extend the photocatalytic effectiveness of ZnO to the visible domain, several experimental approaches have been explored including doping with metals and/or non-metals, dye sensitization, and mixing ZnO with other metal oxides [5,15,16,17].Natural materials such as chitosan and bentonite have retained special interest of researchers. Chitosan, which is a biopolymer, exhibits excellent adsorptive properties towards dyes and heavy metals [18,19,20,21,22] because of the presence of amino (-NH_2_) and hydroxyl (-OH) groups which act as adsorption sites. In addition, chitosan is widely used in food preservation, cosmetics, drug-delivery systems, and remediation of wastewater [23]. However, the low thermal stability and poor mechanical property limit its application [23]. These shortcomings could be overcome by the incorporation of clay in the biopolymer matrix [24,25]. Bentonite is a type of clay possessing a large amount of hydroxyl groups and exhibiting a good thermal and mechanical stability. It is often used as an adsorbent for azo-dyes [26]. Many published works reported on the use of clay/chitosan and ZnO/chitosan composites as adsorbents or photocatalysts in removing dyes [5,13,24,25,27,28]. Fatimah et al. [13] reported that the insertion of ZnO in monmorillonite matrix improved the band gap energy and enhanced the rate of photooxidation of methylene blue.In a previous investigation, Aadnan et al. [5] indicated that the photocatalytic performance under visible light was significantly improved by adding 7 wt% of ZnO to the chitosan biomaterial. Therefore, the challenge we are facing isto extend the photocatalytic activity of ZnO to the visible domain by using eco-friendly materials while improving the thermal stability of the synthesized hybrid-biocomposite. The main goals of this study are: (1) The synthesis of a hybrid-biocomposite “ZnO-Bentonite/Chitosan” using low cost and environmentally friendly materials (Bentonite and chitosan) and ZnO. (2) The study of the structural, optical, thermal, and morphological properties of the prepared hybrid-biocomposite. (3) The study of the photocatalytic performance in degradation of Methyl Orange (MO). MO was used in this study as a molecule probe because it is commonly used as an azo-dye pollutant in numerous investigations. In addition, it presents serious environmental hazards because of its wide use in textile, printing, paper manufacturing, pharmaceutical, and food industries [27]. The raw natural bentonite used in this work was taken from a deposit in the Northeast of Morocco, and purified by sedimentation following the experimental procedure described in reference [26]. The chitosan “CS” was obtained from shrimp shells which collected from the fishmongers in Meknes city (Morocco), following the procedure described in reference [5]. ZnO nanoparticles were synthesized using a precipitation method following the procedure described in reference [8]. The hybrid-biocomposite was characterized using various techniques such as the X-ray diffraction (XRD), Fourier transform infrared (FTIR), UV-vis diffuse reflectance spectroscopy (DRS), thermogravimetric Analysis (TGA), X-ray photoelectron spectroscopy (XPS), and scanning electron microscopy coupled with energy dispersive spectroscopy (SEM-EDS). The photocatalytic efficiency of the synthesized hybrid-biocompositetowards MO was evaluated under both UV-A and visible light irradiation.

## 2. Materials and Methods

### 2.1. Materials

Zinc acetate dihydrate (Zn(CH_3_COO)_2_, 2H_2_O; purity > 99.99%), acetone (C_3_H_6_O; purity > 98.08%) and acetic acid (CH₃COOH; purity > 98%) were purchased from Sigma Aldrich Chemicals (St. Louis, MO, USA). Sodium hydroxide (NaOH; purity > 98%) was purchased from Fisher Scientific International Company (Hampton, VA, USA). Methyl orange (C_14_H_14_N_3_NaO_3_S; purity > 95%) was purchased from Loba Chemie reagents (Mumbai, Maharashtra, India). The natural bentonite material “PB” used in this work was purified by sedimentation following the experimental procedure described in reference [26]. It is composed of SiO_2_ (54.80%), Al_2_O_3_ (18%), Fe_2_O_3_ (6%), MgO (5.12%), Na_2_O (1.75%), and other oxides in traces. The procedure of the preparation of chitosan “CS” is described in detail in reference [5]. Distilled water was used in all experiments.

### 2.2. Synthesis of the Materials

#### 2.2.1. ZnO Nanoparticles

The ZnO nanoparticles were prepared using the precipitation method following the procedure described by El Mragui et al. [8]. The required amount of zinc acetate was dissolved in a 100 mL of distilled water. Then, 20 mL of an aqueous NaOH solution (1 M) was added dropwise under 50 °C during 60 min. The obtained suspension was centrifuged, a white precipitate was collected, and then washed until the pH comes neutral. The obtained precipitate was dried at 100 °C overnight, then ground and calcined at 500 °C for 3 h.

#### 2.2.2. Bentonite/Chitosan “PB/CS”

The synthesis of the PB/CS biocomposite was done as follows. An amount of chitosan was dissolved in an aqueous acetic acid solution (1.5% *v*/*v*). Simultaneously, a quantity of PB, corresponding to 7 wt% in the final PB/CS biocomposite, was dispersed in 100 mL of distillated water, and then added drop wise to the suspension of chitosan. The mixture was stirred continuously at 50 °C for 24 h. The resulting material was centrifuged, washed with distilled water, and then dried at 55 °C for three days.

#### 2.2.3. ZnO-Bentonite/Chitosan “Zn-PB/CS”

The ZnO-bentonite/chitosan hybrid-biocomposite was synthesized in two steps. The first one was dedicated to the preparation of ZnO/Bentonite material containing 7 wt% of ZnO basing on the results reported by Fatimah et al. [13]. These authors showed the insertion of 5 wt% of ZnO in montmorillonite matrix increased adsorption capability of ZnO/montmorillonite and helped to enhance the rate of photooxidation reaction. During this step, an aqueous suspension of PB was prepared by dispersing the necessary amount of PB in 100 mL of water and stirred for 12 h. Then, a suspension of ZnO containing the desired amount of ZnO was prepared and added drop wise to the PB suspension, while stirring at 50 °C for 1 h. The second step concerned the synthesis of the hybrid-biocomposite respecting 7 wt% of Zn-PB. The used of weight ratio was based on a previous study [5] which reported that the optimal photocatalytic performance of ZnO/Chitosan composite was achieved by adding 7 wt% ZnO to chitosan biomaterial. Other study [29] reported that the biocomposite containing 5 wt% of bentonite provided the better thermal and adsorptive properties. The preparation procedure consisted of adding the resulting mixture from the first step drop wise to 250 mL of an aqueous acetic acid solution (1.5 *v*/*v*) containing CS. The obtained mixture was stirred at 50 °C for 24 h, and then centrifuged. The resulting hybrid-biocomposite (Zn-PB/CS), containing theoretically 0.5 wt% of ZnO, 6.5 wt% of PB, and 93 wt% of CS, was washed and dried at 55 °C for three days.

### 2.3. Characterization

The crystalline structure of the materials was analyzed by powder X-ray diffraction (XRD) using a X’PERT MPD_PRO Diffractometer (Malvern Panalytical Ltd., Malvern, United Kingdom) with Cu Kα radiation at 45 kV and 40 mA (λ = 1.5406 Å). FTIR spectra of the samples were recorded from 400–4000 cm^−1^ using a FTIR spectrometer type JASCO 4100 (Jasco International, Tokyo, Japan) and the KBr pellet method. The scanning speed was 2 mm/sand 40 scans were accumulated with 4 cm^−1^ as a resolution. The UV-vis diffuse reflectance spectroscopy (DRS) measurements were made on a JASCOV-570 spectrophotometer (Jasco International, Tokyo, Japan) equipped with a Labsphere DRA-CA-30I integration sphere using BaSO_4_ as a reference. The Differential Thermal Analysis (DTA) was performed between 20 and 400 °C under a stream of air using a Shimadzu Simultaneous DTA-TG apparatus (DTG-60) (Kyoto, Japan). The heating rate was 10 °C/min. The chemical states of the elements were determined by the X-ray photoelectron spectroscopy (XPS) analysis using a Kratos AXIS Ultra HAS equipment (Kyoto, Japan). The analysis was performed with a monochromatic Al Kα X-ray source (1486.7 eV). The morphology and the chemical composition of the materials were obtained by scanning electron microscopy (Quanta 200 from FEI Company, Hillsboro, Oregon, USA) coupled with energy dispersive spectroscopy (SEM-EDS). The sample in the form of a powder was deposited onto a sample holder, and all loose particles from the sample were removed by spraying dry air on the sample. Then, the sample holder was introduced into the microscope for analysis.

### 2.4. Pollutant Removal

The photocatalytic effectiveness of the synthesized composite was assessed using methyl orange as a probe molecule pollutant. The photocatalytic reactions were carried out at room temperature (26 ± 2 °C) and pH 4 in a cylindrical beaker containing an aqueous suspension (10^−5^ M of MO and 0.5 g L^−1^ of hybrid-biocomposite). The suspension was stirred using a magnetic stirrer in the dark for the necessary time to obtain the adsorption/desorption equilibrium. After that, the UV or visible lamp was turned on to initiate the photocatalytic reaction. Test samples were withdrawn at given times of reaction and filtered through a 0.45 µm Millipore filter. The MO concentration monitoring was carried out by measuring the absorbance at λ_max_ = 465 nm using a UV-vis spectrophotometer (Shimadzu 2100 spectrophotometer). A low-pressure lamp (40 W, model Vilber, VL-340.BL, Eberhardzell, Germany) emitting UV radiation at 365 nm (light intensity ≈ 413 mW cm^−2^) and a commercial Feit White Compact Fluorescent lamp (23 W, cool daylight, 6500 K, 1311 Lumens, Mainhouse Electronic Co., Ltd., Xiamen, China) were used to produce UV-A and visible-light irradiations, respectively. The reactor was positioned at about 10 cm below the light source. When the visible lamp was used, the UV radiations were eliminated by placing between the reactor and the light source a chemical filter composed of an aqueous solution of sodium nitrite (0.73 M) [5]. The MO removal percentage was calculated using the following equation:

MO removal (%) = 100 × (C_0_ − C_t_)/C_0_, where C_0_ and C_t_ are the MO concentrations at the initial and t time of reaction, respectively.

## 3. Results

### 3.1. Characterization

#### 3.1.1. X-ray Diffraction

**Figure 1:**
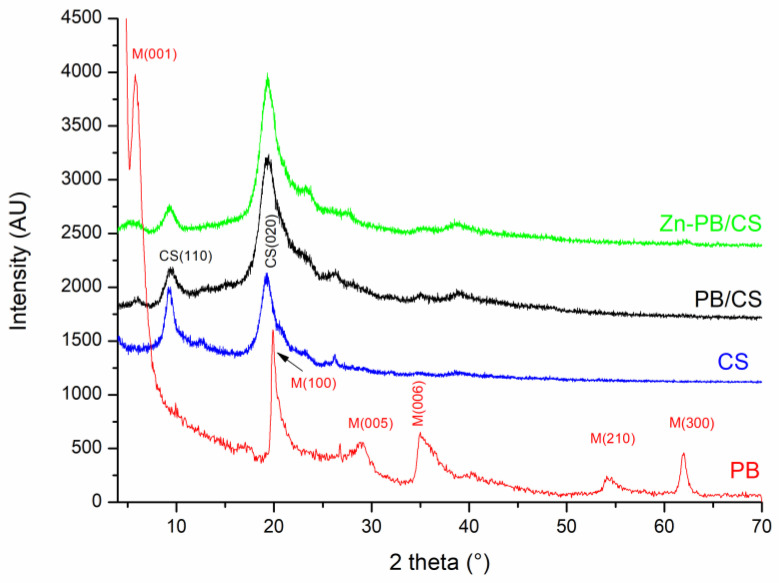
X-ray patterns of the CS, PB, and PB/CS and Zn-PB/CS samples.

The obtained XRD patterns are shown in Figure 1. The broad peaks appearing on the CS spectrum at 2θ = 9.35 and 19.25° match well with those reported previously for CS biomaterial [5], and no further peaks are observed. The broadening of the peaks is due to the low crystallinity of the chitosan. The XRD pattern of PB sample displays peaks belonging to the montmorillonite structure (indicated by M on the spectrum of Figure 1) [26]. The intense peak at 2θ = 5.73° corresponds to the interlamellar distance d_001_= 15.42 Å. The XRD patterns of PB/CS and Zn-PB/CS biocomposites show the two characteristic peaks of CS biomaterial (2θ = 9.35 and 19.25°) as well as a weak peak at 2θ = 5.73° belonging to montmorillonite (d_001_). Compared with XRD pattern of ZnO (Figure S1), no peak of ZnO was observed for Zn-PB/CS hybrid-biocomposite. This is probably due to the very low amount of ZnO and/or the well intercalation of the ZnO in the interlayer of bentonite. On the other hand, the comparison of the XRD spectra of PB/CS and Zn-PB/CS shows that the intensity of the CS peak (d_020_) increases while that of CS (d_110_) decreases significantly. This behavior suggests that the peak appearing at 2θ = 19.25° for PB/CS and Zn-PB/CS is due to the overlap of the peaks of CS (d_020_) [5] and bentonite (d_100_) [5,26] as reported in literature [27,30].

#### 3.1.2. FTIR Spectroscopy

**Figure 2:**
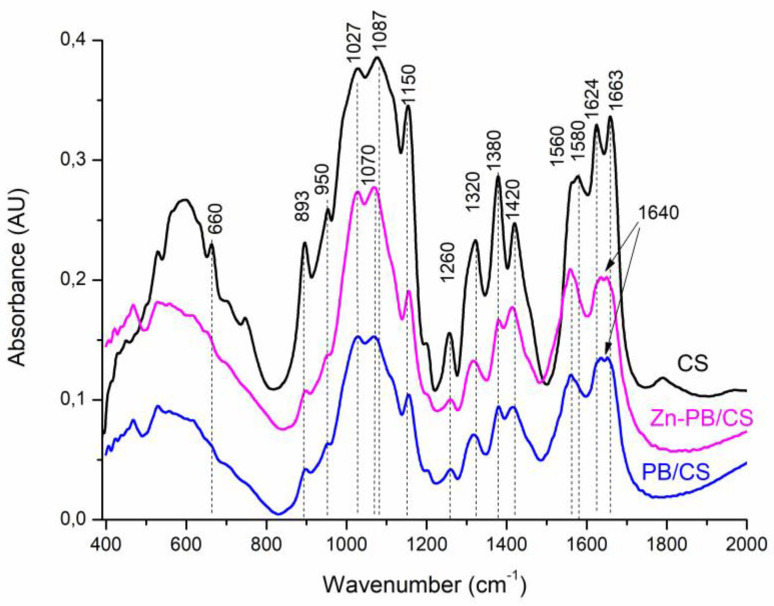
FTIR spectra of CS, PB/CS and Zn-PB/CS biocomposites.

The FTIR spectra of the synthesized materials are shown in Figure S2, Figure 2 and Figure S3. The major characteristic bands can be assigned to the stretching vibrations of O-H and N-H (3460 cm^−1^) [5,27], the stretching vibration of C-H in -CH_2_ and -CH_3_ (2894 and 2927 cm^−1^) [5], the vibration of carbonyl groups in amide I (1663 cm^−1^) [5], the vibration of protonated amino groups (1580 cm^−1^) [5], the stretching vibration of C–N in amide (III) (1380 cm^−1^) [5], the bending vibration of C-H in –CH_2_ (1320 cm^−1^) and CH_3_ (1420 cm^−1^) [5]. The FTIR spectrum of ZnO (Figure S2) shows the characteristic absorption bands of ZnOwürtzite appearing between 400 and 510 cm^−1^ others at about 1425 and 1545 cm^−1^ belonging to C–O and C=O stretching vibrations in acetate groups [9]. The FTIR spectrum of PB (Figure S3) shows bands around 3430 cm^−1^ and 1640 cm^−1^ associated respectively to the stretching and bending vibrations of OH in H_2_O adsorbed on the surface and between the interlayers [26]. The band at 3630 cm^−1^ is attributed to the stretching vibration of OH in (Al,Al)-OH, (Al,Mg)-OH or (Al,Fe)-OH [26]. The bands at 915 cm^−1^ and 880 cm^−1^ are assigned to the bending vibration of the OH group in (Al,Al)-OH and (Al,Fe)-OH, respectively [26] ]. The intense broad band centered at 1038 cm^−1^ is assigned to the Si-O vibration of the tetrahedral sheet [26]. The absorption band at 624 cm^−1^ is related to the perpendicular vibration of the octahedral cations (R-O-Si) where R = Al, Mg, or Fe [26]. The band at 530 cm^−1^ is attributed to the bending vibration of Si–O in Si–O–Al [26]. The band 466 cm^−1^ is assigned to Si-O-Fe and/or Si–O–Al vibrations [26]. The comparison of the FTIR spectra of PB, PB/CS and Zn-PB/CS (Figure 2) indicates clearly that all the characteristic bands of PB decrease in the intensity or disappear for PB/CS and Zn-PB/CS samples, particularly those of Si-O (1038 cm^−1^) and (Al,Al)-OH (3630 cm^−1^) vibrations. On the other hand, the comparison of the spectra of the samples containing chitosan (Figure 2) reveals a significant decrease in the intensity of the bands related to the C–OH stretching vibration (at 1150 cm^−1^) [31], the C–N stretching vibration of the amide III (at 1380 cm^−1^), and the protonated amino group (at 1580 cm^−1^). Furthermore, the band at 1663 cm^−1^ belonging to the carbonyl groups in amide I which shifts to 1640 cm^−1^ suggests that -NH_2_ and -OH groups interact with ZnO [5] and PB [32]. The band at 1087 cm^−1^ belonging to the secondary -OH of chitosan which shifts to 1070 cm^−1^ indicates the existence of a coordination of -OH groups with ZnO [5]. Therefore, the significant decrease of the intensity of the characteristic bands of both PB and CS, and the shift of some characteristic bands of chitosan suggest the establishment of strong interactions between the groups of chitosan (C–O, C=O, -NH_2_, OH) and the aluminosilicate structure of bentonite (Al^3+^ and Si^4+^) on the one side, and between the functional groups of chitosan (-NH_2_, OH) and ZnO on the other side. Analogous results were reported about the complexation of ZnO with CS by several authors [5,33].

#### 3.1.3. UV-Vis Diffuse Reflectance Spectroscopy

**Figure 3:**
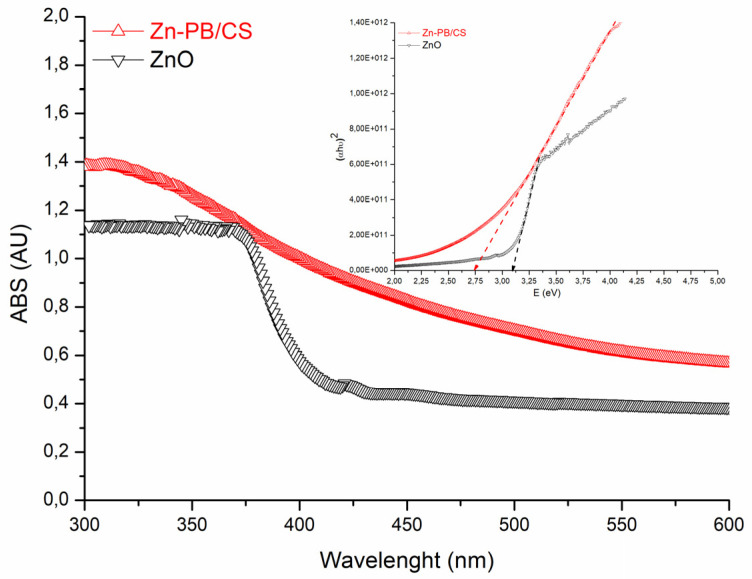
DRS spectra of ZnO and Zn-PB/CS materials.

The DRS spectra of ZnO and Zn-PB/CS are shown in Figure 3. It is clearly seen that the analyzed samples present a good absorption in the UV region with a net improvement for Zn-PB/CS hybrid-biocomposite (about 22%). In the visible domain, ZnO becomes practically transparent while Zn-PB/CS exhibits a long tail, extending thus its absorption up to 500 nm. Based on these results, it seems that the interaction established between chitosan, bentonite and ZnO particles makes the hybrid-biocomposite particles sensitive to visible light. Similar results were reported by Aadnan et al. [5] and Farzana et al. [11] concerning the ZnO-Chitosan biocomposite. The estimated band gap energy (Eg) values of the as-prepared samples were obtained by plotting (αhν)^2^ versus hν (inset of Figure 3), assuming an indirect band gap transition for ZnO [5,8]. The obtained Eg value for ZnO (3.12 eV) indicates that ZnO cannot absorb wavelengths above to about 400 nm, whereas that of Zn-PB/CS (2.73 eV) suggests that the hybrid-biocomposite becomes sensitive to visible light. Thereby, an improvement of the photocatalytic efficiency of Zn-PB/CS sample under visible light is expected.

#### 3.1.4. Thermogravimetric and Differential Thermal Analyses

**Figure 4:**
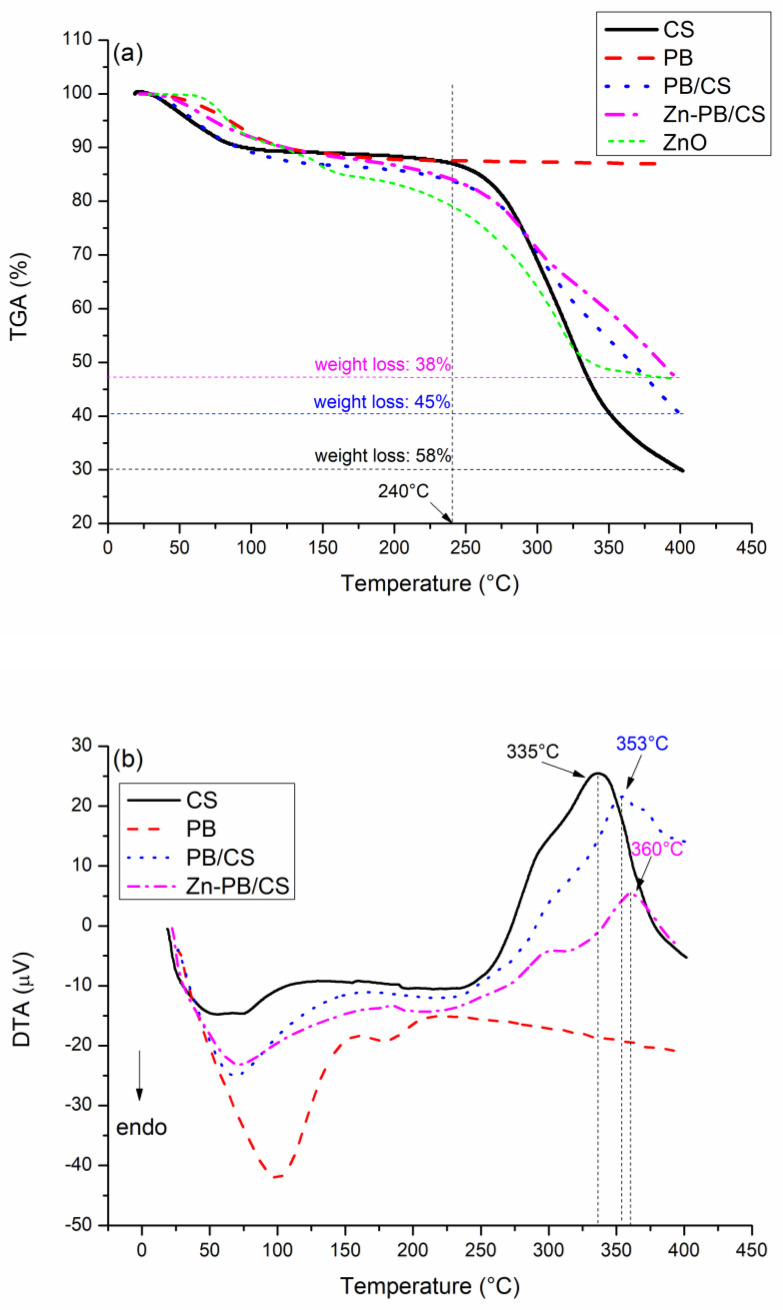
(**a**) Thermogravimetric and (**b**) differential thermal analyses curves of CS, PB, PB/CS, and Zn-PB/CS samples.

The thermogravimetric curve of Zn-PB/CS along with those of CS, PB, ZnO, and PB/CS samples are shown in Figure 4a. The Thermogravimetric curves of ZnO and samples containing CS exhibit two main weight loss regions. The first one is before 240 °C. It is accompanied with endothermic peaks (Figure 4b). These peaks are due to the evaporation of adsorbed water as well as the residual solvents [34], validating the FTIR results. The second is above 240 °C. It corresponds to the thermal decompositionof CS, as well as to the acetate groups linked to Zn [5,11,34] since a wide exothermic peak is observed for all samples containing CS (Figure 4b). It is noteworthy that the maximum temperature of the decomposition of CS increases progressively from 335 °C (for CS) to 353 °C (for PB/CS), and then to 360 °C (for Zn-PB/CS) (Figure 4b). This behavior proves experimentally that the decomposition of CS was postponed progressively by adding PB and ZnO, and the thermal stability of the synthesized Zn-PB/CS hybrid-biocomposite was improved.

The comparison of the weight losses observed above 240 °C for CS (58%), PB/CS (45%), ZnO (31%) and Zn-PB/CS (38%) (Figure 4a) suggests that ZnO and PB particles tend to hinder the thermal decomposition of CS.Therefore, the thermal stability of PB/CS and Zn-PB/CS biocomposites is improved. Based on these results, it is allowable to suggest the existence of strong interactions between the groups of chitosan (C–O, C=O, -NH_2_, OH) and the ZnO nanoparticles and bentonite. Analogous results were reported by Aadnan et al. [5] who indicated that the incorporation of an optimum of ZnO into chitosan polymer creates a strong interaction between the amino and hydroxyl groups of chitosan and Zn^2+^. In addition, Hristodor et al. [35] and Kausar et al. [36] suggested that the intercalation of CS in clay allowed a strong interaction between the amino and hydroxyl functional groups of CS and the silicate layer of clay.

The TGA analysis of PB sample records a 13% weight loss between 25 and 400 °C (Figure 4a) accompanied with a wide endothermic peak (Figure 4b). This behavior is assigned to the evaporation of water molecules adsorbed onto the surface and/or into the interlayers. This is probably associated with the dehydration phenomena of exchangeable cations [37].

#### 3.1.5. X-ray Photoelectron Spectroscopy

**Figure 5:**
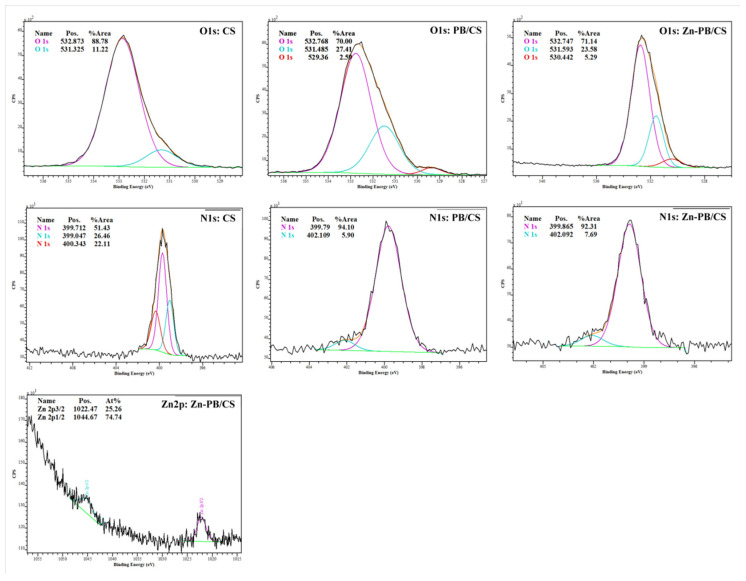
High-resolution XPS spectra of O1s, N1s and Zn2p in the synthesized biocomposites.

Figure S4 gives the full scan XPS spectra of the prepared materials in which the main observed peaks (O1s, C1s, N1s, Zn2p, Al2p, and Si2p) were identified. The high-resolution spectra of the O1s, N1s and Zn2p signals are shown in Figure 5, while those of C1s, Al2p and Si2p are given in Figures S5–S7, respectively.

**Scheme 1:**
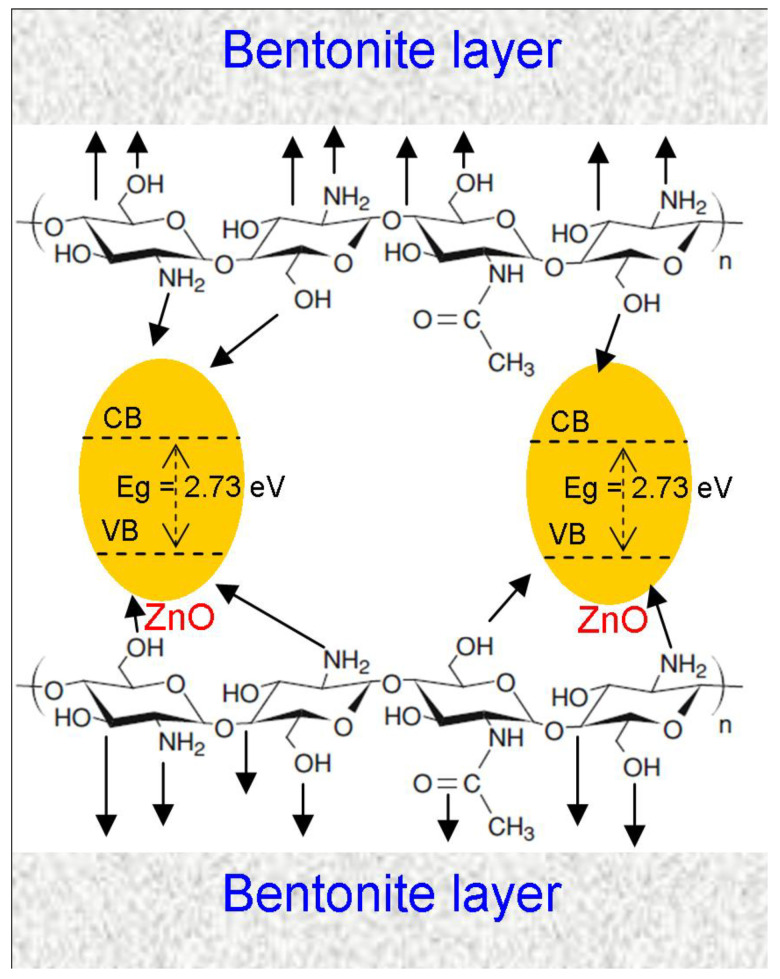
Schematic illustration of the interaction between the ZnO nanoparticles, the functional groups of chitosan, and the alumina-silica sheets of bentonite.

The full scan testifies the presence of Al2p at 75 eV and Si2p at 103 eV [38,39] for PB/CS and Zn-PB/CS biocomposites. The deconvolution of O1s spectrum of CS (Figure 5) reveals the contribution of two peaks at 531.32 and 532.8 eV, which could be linked to C=O bond and OH, and/or >C–O bonds [40]. On the high-resolution spectra of PB/CS and Zn-PB/CS, the intensity of the peak at about 532.8 eV decreases, while that at 531.3 eV increases slightly because of the contribution of the bending energy due to Si–O–Si and Al-(OH) coming from the aluminosilicate structure of bentonite [41]. Furthermore, a third peak at about 530 eV is observed for the biocomposites containing bentonite, which is attributed to the Si–O–Si, Si–O–Al bonds in PB/CS, Zn-PB/CS [41], and/or Zn–O bond in the Zn-PB/CS hybrid-biocomposite [5,42]. The deconvolution of N1s spectrum of CS reveals the contribution of three peaks at 399.04, 399.71, and 400.34 eV, which could be attributed to the –NH-, -NH_2_ and -NH_3_^+^ groups [5,40,43], confirming the FTIR results. The high resolution of N1s spectra of the BP/CS and the Zn-PB/CS samples shows a slight shift of the characteristic peaks of -NH_2_ and -NH_3_^+^ groups with a significant variation in their corresponding intensities. This is an indication that the functional groups of chitosan interact strongly with both ZnO [5] and the structure of bentonite as highlighted by the FTIR, XRD, and thermal measurements. The binding energies at about 1022 and 1047 eV are attributed to Zn2p3/2 and Zn2p1/2, respectively [5], validating the presence of Zn2+ state in Zn-PB/CS sample. The deconvolution of the XPS spectra of C1s (Figure S5) obtained for the samples containing CS highlights the contribution of three peaks. The first one at about 285 eV is attributed to C-C (sp3) bonds [43,44]; the second at 286 eV is associated to C–O and/or C=O groups [43,44]; and the third at about 289 eV is assigned to C=O and/or O-C–O bonds [43,44]. Compared with the spectrum of CS, it is clearly observed that the intensity of the peak at 285 eV increases for PB/CS and Zn-PB/CS while for the peak at 286 eV, an opposite behavior is observed. In the meanwhile, the intensity of the peak at 289 eV remains practically unchanged. This suggests that the C–O and C=O groups of chitosan interact with the aluminosilicate structure of bentonite. Figure S6 shows that the position of the peak of Al2p (74.7 eV) for PB/CS and Zn-PB/CS is slightly lower than for PB (75.06 eV). A similar observation is made for the peak of Si2p (Figure S7) which decreases slightly from 103.1 eV (for PB) to 102.8 eV (for PB/CS and Zn-PB/CS). All of these results supported with those obtained by XRD, FTIR and thermal measurements indicate clearly the existence of strong interactions between the C–O, C=O, -NH_2_, and OH groups of chitosan and the alumina-silica sheets of bentonite on the one side, and between the chitosan’s functional groups (-NH_2_, OH) and ZnO on the other side. Scheme 1 illustrates the interactions which exist between ZnO nanoparticles, the functional groups of chitosan, and bentonite in the hybrid-biocomposite system. In this system, it is very plausible to assume that ZnO nanoparticles interact with –NH_2_ and –OH groups of chitosan [5,33] in order to form a complex between ZnO and Chitosan as indicated by the XRD, FTIR, and XPS results. On the other hand, chitosan polymer interacts with the alumina-silica sheets of bentonite via the C–O, C=O, -NH_2_, and OH groups as highlighted by the XPS analysis.

#### 3.1.6. SEM-EDS

**Figure 6:**
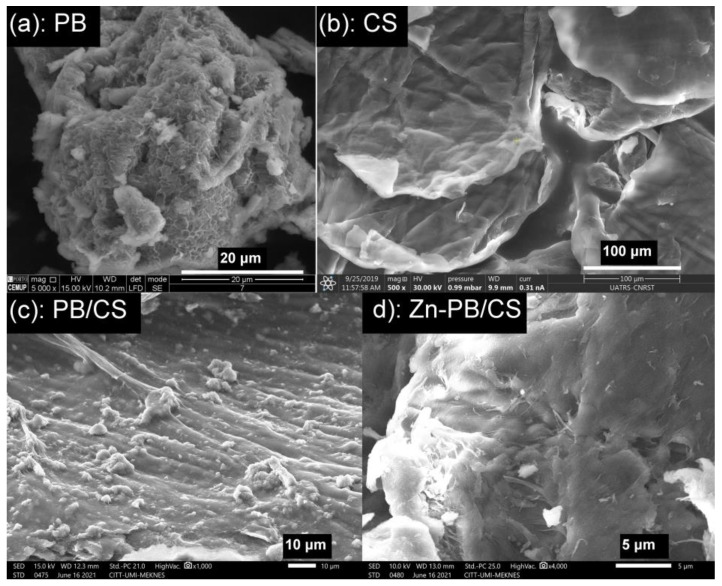
SEM micrographs of (**a**) PB, (**b**) CS, (**c**) PB/CS and (**d**) Zn-PB/CS samples.

Figure 6a–d depicts the SEM images of PB, CS, PB/CS and Zn-PB/CS samples respectively. Figure 6a shows homogeneous particles of PB in flaky form. The CS sample (Figure 6b) is composed of crooked lamellar particles with homogeneous, smooth, and compact surface. The EDS analysis indicates the presence of C, O and N (Figure S8). From Figure 6c,d, it can be observed that the surface of PB/CS and Zn-PB/CS hybrid-biocomposites becomes less compact and rougher by comparison with CS, and presents heterogeneous structures with holes and fractures. It is easy to see the presence of bentoniteparticles which are scattered onto the surface. The EDS spectra of PB/CS and Zn-PB/CS (Figure S8) highlight the presence of the different oxides of bentonite for PB and PB/CS samples along with ZnO for Zn-PB/CS sample. Moreover, the EDS mapping analyses (Figure S9) shows clearly the homogeneous dispersion of Zn on the surface of Zn-PB/CS hybrid-biocomposite, promoting an interaction between ZnO and bentonite and chitosan.

### 3.2. Photocatalytic Tests

**Figure 7:**
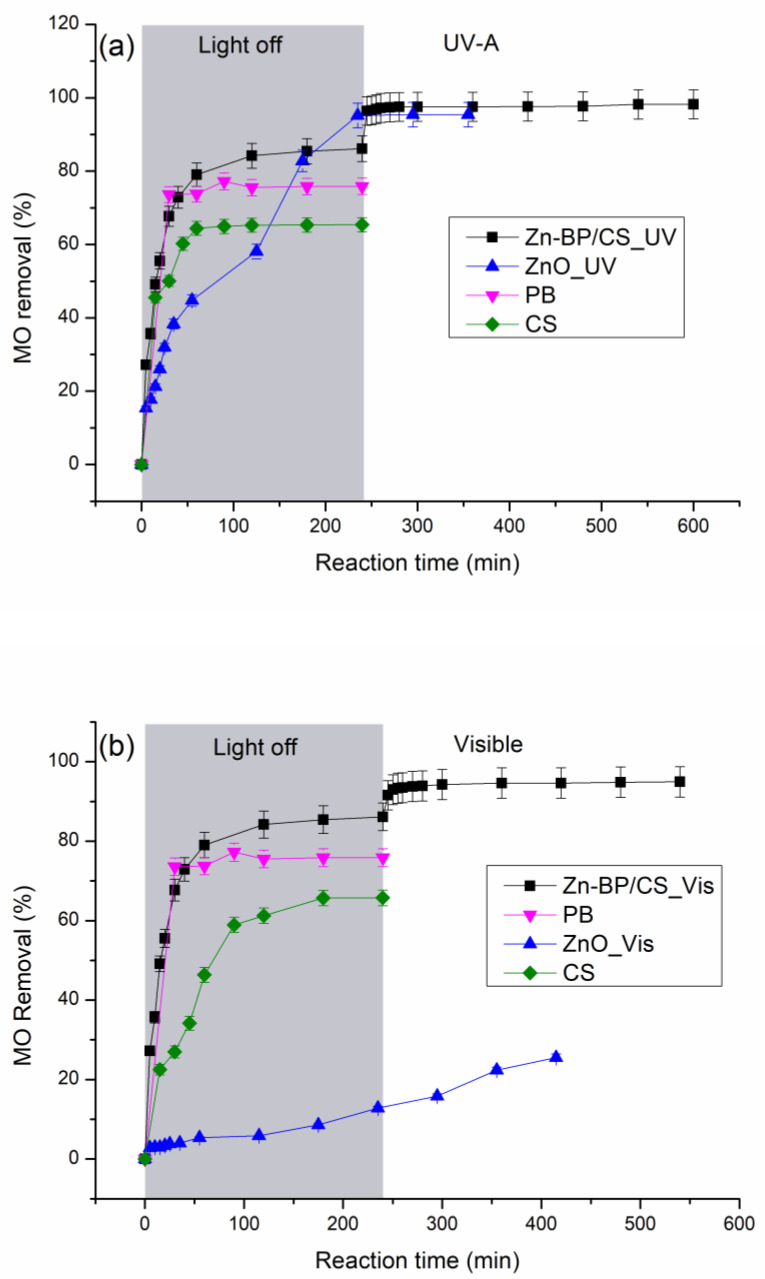
MO removal as a function of irradiation time under (**a**) UV-A light and (**b**) visible light.

The photocatalytic efficiency of the prepared hybrid-biocomposite was evaluated by using methyl orange as a molecule probe. In order to have a fair evaluation, experiments were also carried out in the presence of ZnO, CS and PB samples. Before lighting the UV-A or visible lamps, the aqueous suspension containing 0.5 g L^−1^ of ZnO, CS, PB, or Zn-PB/CS was stirred in the dark for the necessary time to achieve the adsorption/desorption equilibrium. Figure 7 shows the MO removal curves as a function of reaction time under UV-A and visible light. From the obtained results in the dark (Figure 7a,b), it appears that the MO removal is significantly improved in the presence of Zn-PB/CS hybrid-biocomposite when compared with ZnO, PB and CS materials. In fact, the final MO removal in the presence of PB (75.8%) and CS (65.4%) increases significantly in the presence of Zn-PB/CS (86.1%). This is probably the result of the strong interactions established between the groups of chitosan (C–O, C=O, -NH_2_, OH) and the aluminosilicate structure of bentonite (Al^3+^ and Si^4+^) on the one side, and between the functional groups of chitosan (-NH_2_, OH) and ZnO on the other side. On the other hand, under UV-A (Figure 7a) and visible light irradiation (Figure 7b), the hybrid-biocomposite Zn-PB/CS exhibits the best final MO conversion (98.2 % under UV-A and 94.2 % under visible light) by comparison with ZnO used as a benchmark (95.4 % under UV-A and 25.5 % under visible light). These results prove clearly that the photocatalytic efficiency of Zn-PB/CS is improved by 3% under UV-A, and 268 % under visible light. This behavior is expected taking into account the DRS results which showed an improvement of the absorbance for Zn-PB/CS hybrid-biocomposite (+22%) by comparison with ZnO nanoparticles. Therefore, the very impressive improvement of MO removal (+268%) observed under visible light could be due to the reduction of the band gap energy (2.73 eV) by comparison with ZnO (3.12 eV). A lot of studies dealing with the photocatalytic performance of various composites containing chitosan modified by ZnO and/or montmorillonite were reported [5,12,28,45]. They indicated that the improvement of the photocatalytic activity of ZnO-chitosan composite could be ascribed to the synergistic effect of both the reduction of the band gap energy and the separation of the charge carriers. In this study, taking into account the XRD, FTIR, DRS, DTA, and XPS results, it is quite legitimate to assume that the best MO conversion obtained under visible light irradiation in the presence of Zn-PB/CS could be attributed to the decrease of the Eg observed for the hybrid-biocomposite as well as to the blocking of the recombination of charge carriers which is promoted by the strong interactions established between the C–O, C=O, -NH_2_, and OH groups of chitosan and the aluminosilicate structure of bentonite on the one side, andbetween the functional groups of chitosan (-NH_2_, OH) and ZnO on the other side.

**Figure 8:**
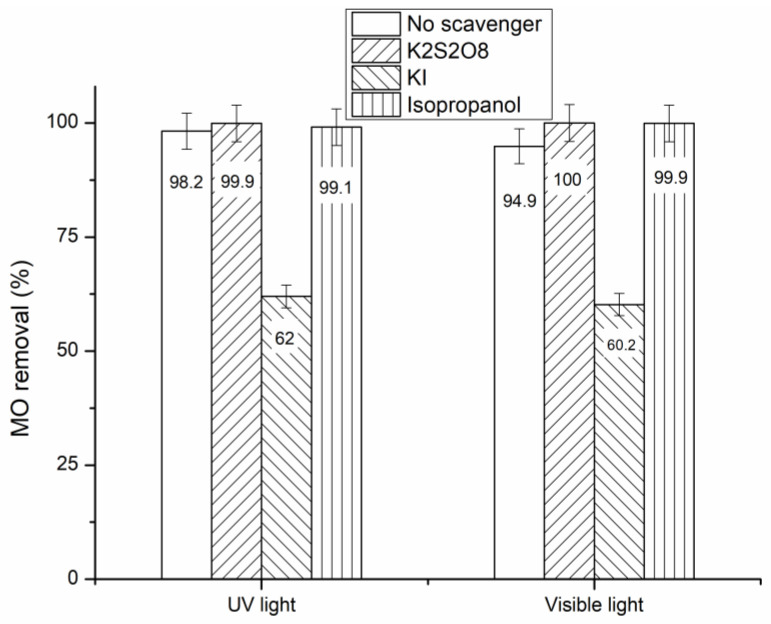
MO conversion obtained for Zn-PB/CS under UV-A and visible light irradiation in the presence of e^−^, h^+^, and HO^•^ scavengers.

In order to identify the role of the primary active species engaged in the degradation of MO under UV-A and visible light for the prepared hybrid-biocomposite, some active species (e^−^, h^+^, HO^•^) scavenging experiments were carried out, and the obtained results were compared with the absence of any scavenger. The active species trapping experiments were conducted using K_2_S_2_O_8_ as an electron scavenger, KI as a hole scavenger and isopropyl alcohol as a hydroxyl radical scavenger [46]. As shown in Figure 8, the addition of electron and hydroxyl radical trappers increases slightly the MO removal under both UV-A and visible light, indicating that these two active species do not play the major role in the photocatalytic process. In contrast, the addition of KI inhibits significantly the MO degradation by about 37% under both UV-A and visible light, indicating clearly that the holes are the main oxidative active species involved in the MO degradation. Analogous results have been reported for ZnO-Chitosan biocomposite used as photocatalyst in the MO degradation under UV and visible light [5]. Therefore, the suggested mechanism of the photocatalytic degradation of MO under UV-A and visible light in the presence of Zn-PB/CS hybride-biocomposite can be described by the following equations:

(1) $$\left(\mathrm{Zn}-\mathrm{PB}/\mathrm{CS}\right)\overset{hv}{\to }\left(\mathrm{Zn}-\mathrm{PB}/\mathrm{CS}\right)+e_{\mathrm{CB}}^{-}+h_{\mathrm{VB}}^{+}$$

(2) $$e_{\text{CB}}^{-}+h_{\mathrm{VB}}^{+}\to \text{heat}$$

(3) $$H_{2}O_{\mathrm{ads}}+h_{\mathrm{VB}}^{+}⇌(H^{+}+{\mathrm{OH}}^{-}{)}_{\mathrm{ads}}+h_{\mathrm{VB}}^{+}\to H^{+}+{\mathrm{OH}}_{\mathrm{ads}}^{•}$$

(4) $${\mathrm{OH}}_{\mathrm{ads}}^{-}+h_{\mathrm{VB}}^{+}\to {\mathrm{OH}}_{\mathrm{ads}}^{•}$$

(5) $${\left(O_{2}\right)}_{\mathrm{ads}}+e_{\mathrm{BC}}^{-}\to O_{2}^{•-}$$

(6) $$O_{2}^{•-}+H^{+}\to {\mathrm{HO}}_{2}^{•}$$

(7) $$2{\mathrm{HO}}_{2}^{•}\to H_{2}O_{2}+O_{2}$$

(8) $$H_{2}O_{2}+e^{-}\to {\mathrm{OH}}^{•}+{\mathrm{OH}}^{-}$$

(9) $${\mathrm{MO}}_{\mathrm{ads}}+h_{\mathrm{VB}}^{+}\to R_{\mathrm{ads}}^{•+}\to \text{degradation products}\;(\text{the main reaction})$$

(10) $${\mathrm{MO}}_{\mathrm{ads}}+{\mathrm{OH}}_{\mathrm{ads}}^{•}\to R_{\mathrm{ads}}^{•}\to \text{degradation products}\;(\text{the main reaction})$$

## 4. Conclusions

In this investigation, a novel hybrid-biocomposite “ZnO-Bentonite/Chitosan” was synthesized using natural materials (Bentonite and chitosan) and ZnO nanoparticles. The eco-friendly hybrid-biomaterial has been used as a photocatalyst for water decontamination. The thermal measurements showed that the decomposition of CS was postponed progressively by adding PB and ZnO, and the thermal stability of the synthesized Zn-PB/CS hybrid-biocomposite was improved. The XRD, FTIR, DRS, XPS, and SEM results highlighted the existence of strong interactions between the C–O, C=O, -NH_2_, and OH groups of chitosan and the aluminosilicate structure of bentonite (Al^3+^ and Si^4+^) on the one side, and between the functional groups of chitosan (-NH_2_, OH) and ZnO on the other side. The experiments carried out in the dark showed that the MO removal was significantly improved in the presence of Zn-PB/CS hybrid-biocomposite (86.1%) by comparison with PB (75.8%) and CS (65.4%) materials. The photocatalytic experiments carried out under visible light showed that the MO removal has been increased 268 times in the presence of Zn-PB/CS. The radical-trapping experiments suggested that the MO photocatalytic degradation under both UV-A and visible light irradiations involved holes as main oxidative active species.

## Data Availability

No new data were created or analyzed in this study. Data sharing is not applicable to this article.

## Supplementary Materials

The following are available online at https://www.mdpi.com/article/10.3390/nano12010102/s1, Figure S1: XRD patterns of ZnO and Zn-PB/CS materials, Figure S2: FTIR spectra of CS, PB/CS, ZnO and Zn-PB/CS biocomposites, Figure S3: FTIR spectrum of PB sample, Figure S4: Full scan XPS spectra of the hybrid-biocomposites accompanied with those of PB, ZnO and CS, Figure S5: High-resolution XPS spectra of C1s, Figure S6: High-resolution XPS spectra of Al2p, Figure S7: High-resolution XPS spectra of Si2p, Figure S8: EDS analyses of PB, CS, PB/CS and Zn-PB/CS samples, Figure S9: EDS mapping analysis of Zn-PB/CS hybrid-biocomposite
