# Supplementary material for: Physicochemical and Photocatalytic Properties under Visible Light of ZnO-Bentonite/Chitosan Hybrid-Biocompositefor Water Remediation
Source: Nanomaterials (Basel). 2021 Dec 29;12(1):102. doi: 10.3390/nano12010102 (PMC8746606; doi:10.3390/nano12010102)
Supplement: Supplementary file 1 [file nanomaterials-12-00102-s001.zip › nanomaterials-1513510-supplementary.pdf]

nanomaterials

Physicochemical and Photocatalytic Properties under Visible Light of ZnO-Bentonite/Chitosan Hybrid-Biocompositefor Water Remediation

Imane Aadnan [1], Omar Zegaoui [1,*], Abderrahim El Mragui [1] and Joaquim Carlos Gomes Esteves da Silva [2]

[1] Research team "Materials and Applied Catalysis: MCA", CBAE Laboratory, URL-CNRST N°13, Faculty of Sciences, Moulay Ismail University of Meknes, Zitoune, Meknès BP. 11201, Morocco; i.aadnan@edu.umi.ac.ma (I.A.); a.elmragui@edu.umi.ac.ma (A.E.M.)

[2] (UP)—Research Center in Chemistry, DGAOT, Faculty of Sciences, University of Porto, Rua do Campo Alegre, s/n, 4169-007 Porto, Portugal; jcsilva@fc.up.pt

* Correspondence: o.zegaoui@umi.ac.ma

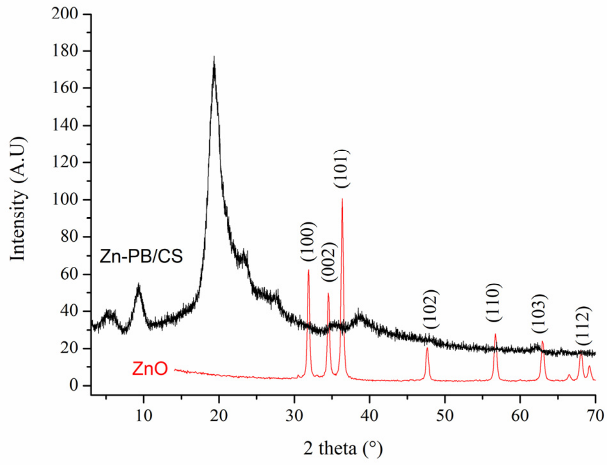

Figure S1. XRD patterns of ZnO and Zn-PB/CS materials.

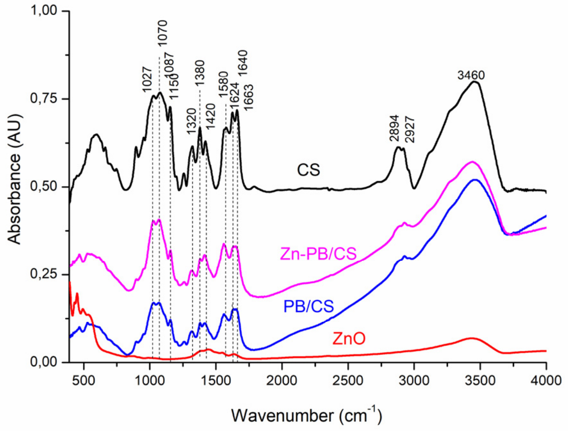

Figure S2. FTIR spectra of CS, PB/CS, ZnO and Zn-PB/CS biocomposites.

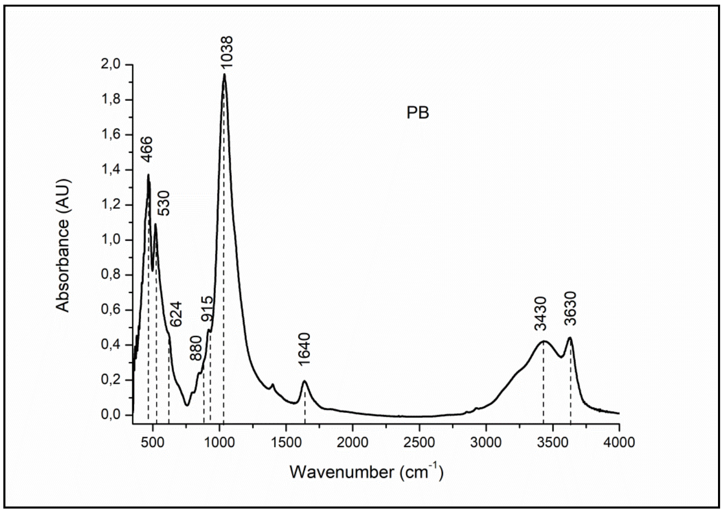

Figure S3. FTIR spectrum of PB sample.

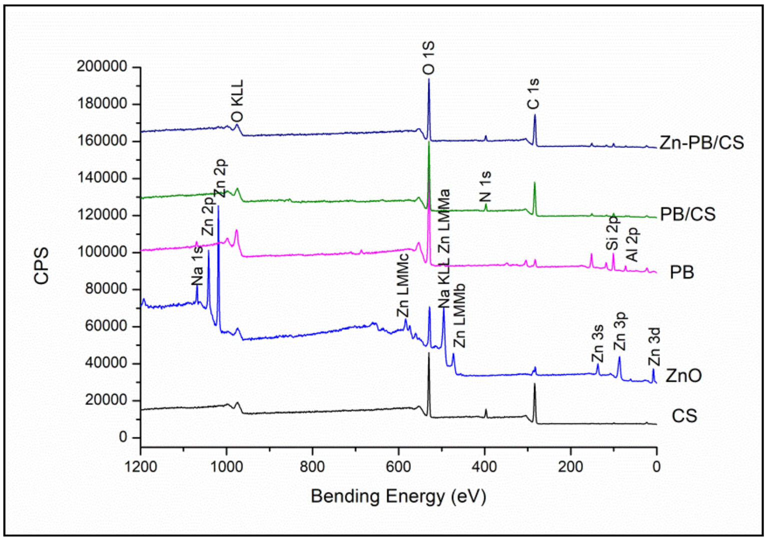

Figure S4. Full scan XPS spectra of the hybrid-biocomposites accompanied with those of PB, ZnO and CS.

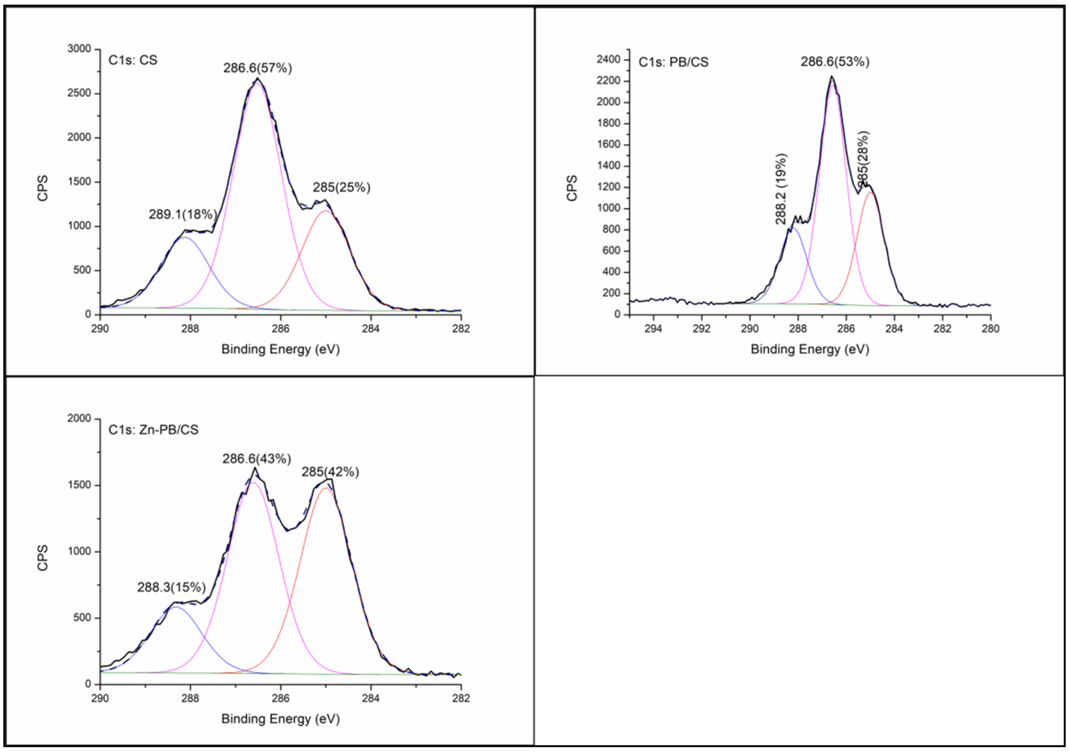

Figure S5. High-resolution XPS spectra of C1s.

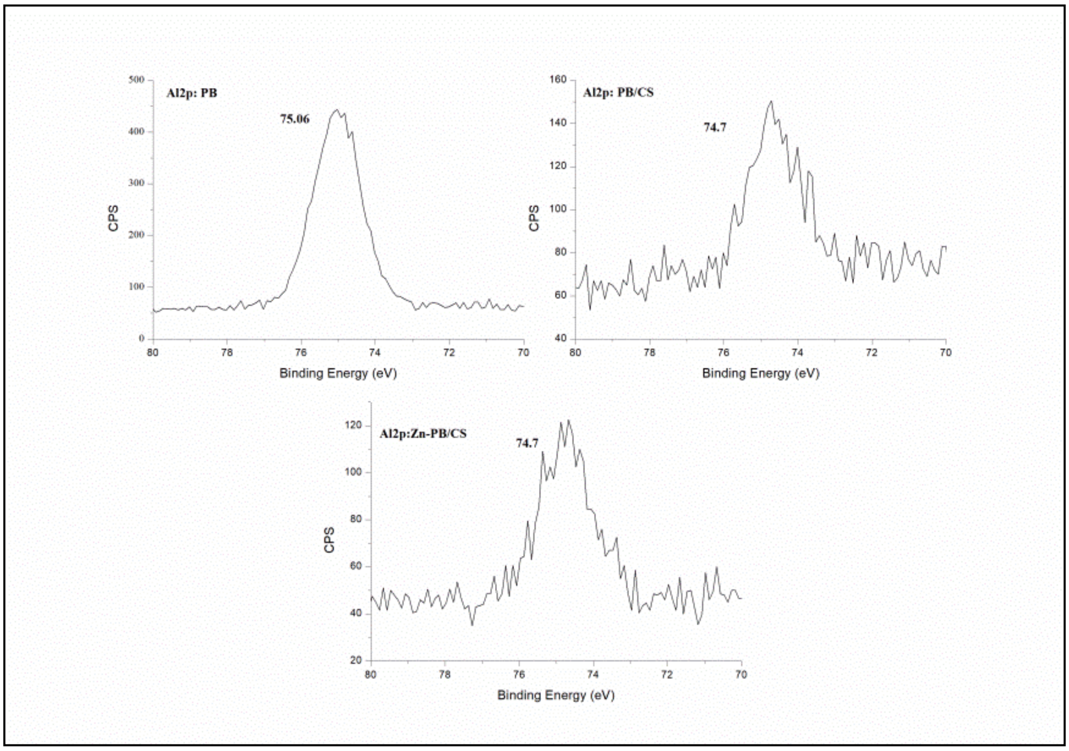

Figure S6. High-resolution XPS spectra of Al2p.

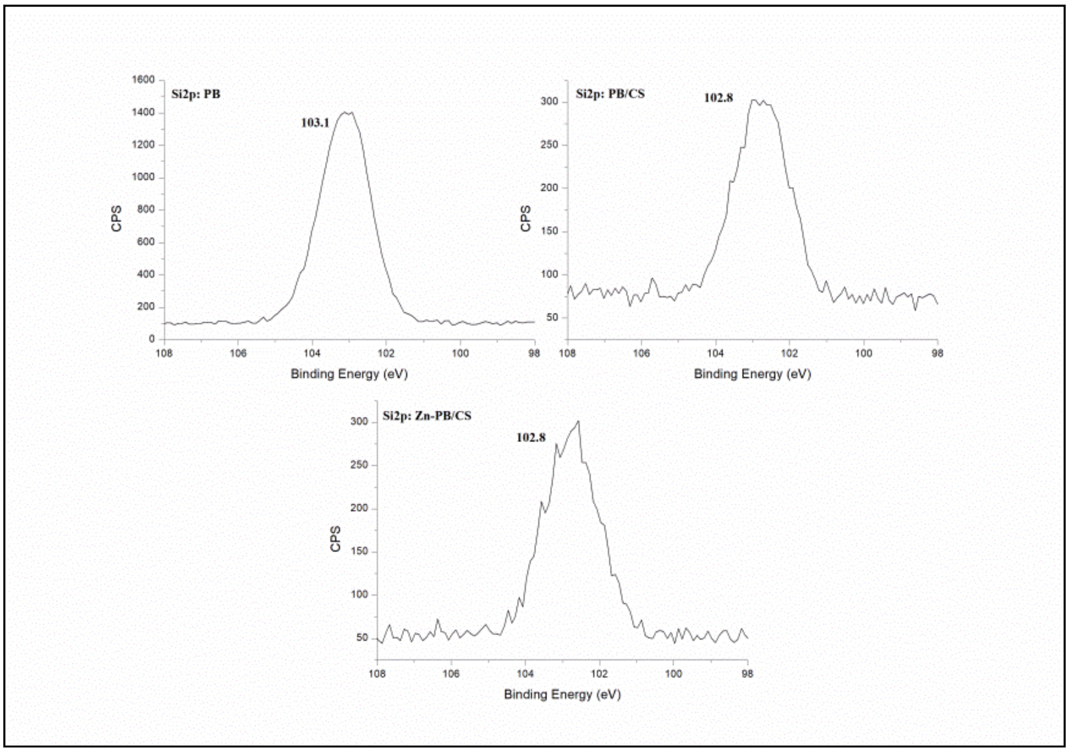

Figure S7. High-resolution XPS spectra of Si2p.

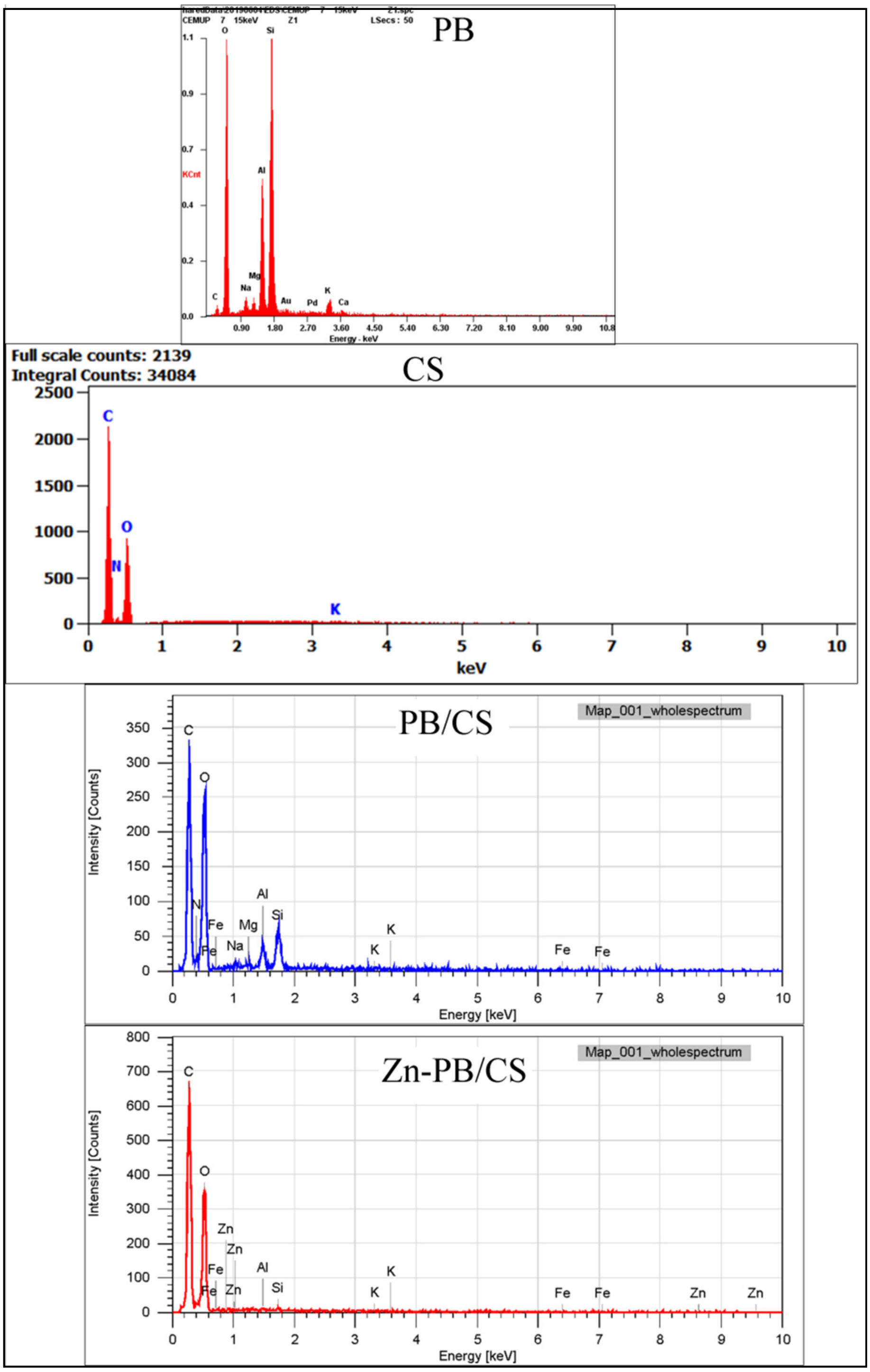

Figure S8. EDS analyses of PB, CS, PB/CS and Zn-PB/CS samples.

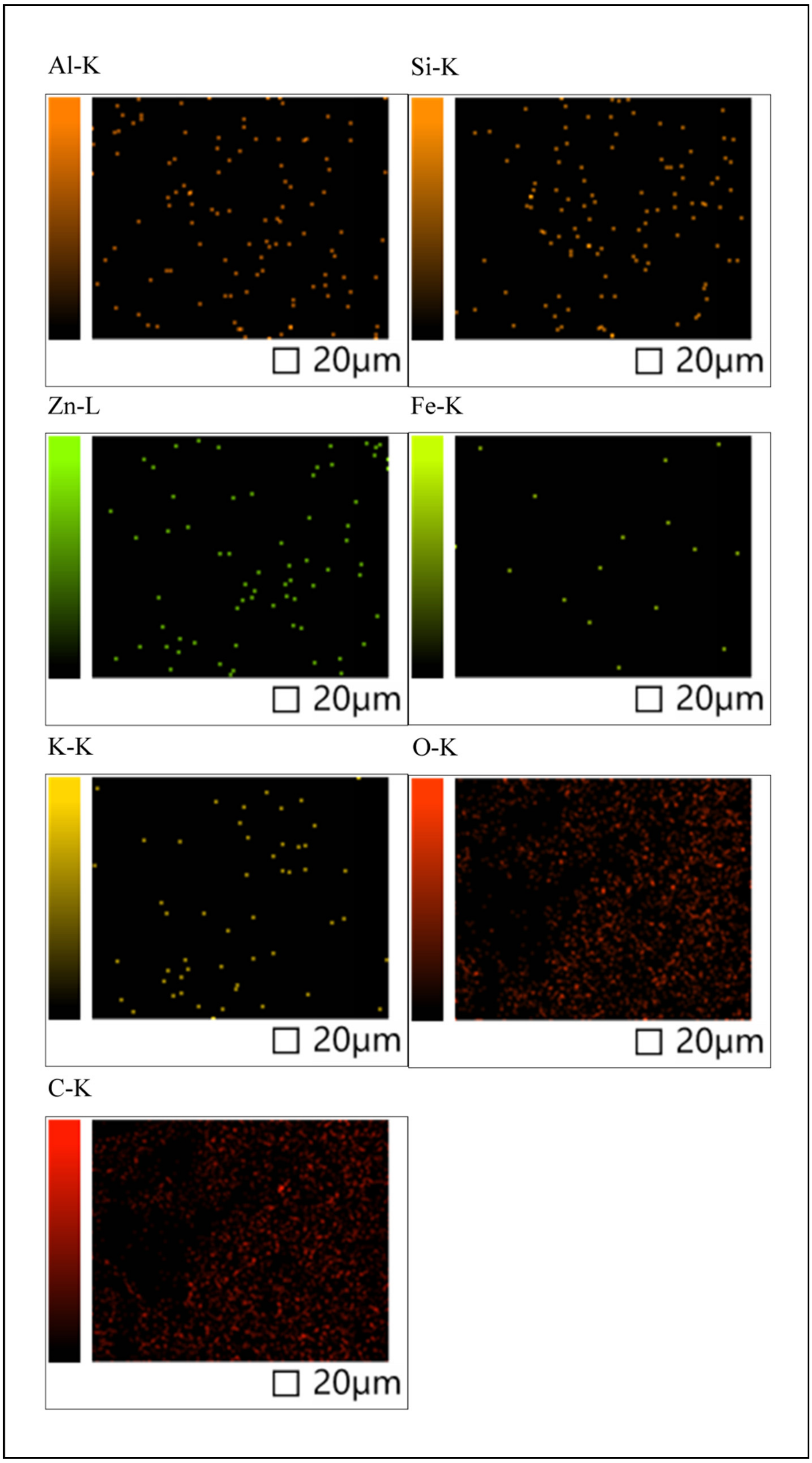

Figure S9. EDS mapping analysis of Zn-PB/CS hybrid-biocomposite.
